# Dr. Mackenzie’s Book

**Published:** 1888-11

**Authors:** 


DR. MACKENZIE'S BOOK.—Dr. Mackenzie has felt himself called upon to write a book in regard to his views and treatment of the Crown Prince of Germany. The editor of the *Medical and Surgical Reporter* makes the following sensible remarks on this matter: "It is not strange that Dr. Mackenzie felt the dissent and distrust of his German associates, while his patient lived, or was irritated at their triumph when the diagnosis which they made and he denied was confirmed, and when the false hopes with which he inspired the victim gave place to despair and death. It would not have been improper if he had, in some brief communication to his professional bretheren, explained the reasons why he so stoutly maintained that the Crown Prince was not suffering with cancer of the larynx, and refused his assent to the extirpation of the growth. In doing this he might even, without incurring much censure, have answered some of the charges and insinuations against him which he attributed to the envy of German medical men. But, unfortunately for his reputation, he has passed beyond the limits to which a discreet man, conscious of being right and confident of the respect of his professional brethren ,would have restricted himself, and has put before the world a statement which will do him more damage than anything which others have stated of him.

*Hydriodic Acid.*—Hypophosphites in Phthisis. Sixth editition, August, 1888. R. W. Gordon, chemist, New York.

